# Single-embryo transfer: a key strategy to reduce the risk for multiple pregnancy in assisted human reproduction

**DOI:** 10.1515/almed-2021-0013

**Published:** 2021-04-02

**Authors:** Pilar Reimundo, Javier M. Gutiérrez Romero, Tamara Rodríguez Pérez, Ernesto Veiga

**Affiliations:** Laboratory of Assisted Reproduction and Andrology, Area of Clinical Biochemistry, Vall d’Hebron Clinical Laboratories, Vall d’Hebron University Hospital, Barcelona, Spain; Clinical Management Unit of Clinical Laboratories, Puerta del Mar University Hospital, Cadiz, Spain; Laboratory of Andrology and Assisted Reproduction Techniques, Service of Clinical Biochemistry, La Paz University Hospital, Madrid, Spain; Unit of Assisted Human Reproduction, Complejo Hospitalario Universitario de Santiago de Compostela (CHUS), SERGAS, Santiago de Compostela, Spain

**Keywords:** assisted reproduction techniques, elective single-embryo transfer, infertility, *in vitro* fertilization, multiple delivery, multiple pregnancy

## Abstract

In the early days of assisted reproductive technology (ART), the main target was achieving gestation. Success rates were low, and multiple embryo transfers became common practice, with multiple pregnancies being 20 times higher than in natural conception. Multiple pregnancy is associated with a higher risk of complications for the mother and the baby than a singleton pregnancy. Added to healthcare costs, multiple pregnancy also involves other costs and psychosocial risks, with a high social and health costs. At present, success rates of assisted human reproduction (AHR) have improved dramatically, partially due to advances in laboratory techniques such as culture of blastocyst-stage embryos and vitrification. Additionally, there is a wide range of counseling, health and economic policies that have demonstrated being effective in increasing single-embryo transfer (SET) practices and reducing multiple pregnancies, which ensures satisfactory success rates. Therefore, single-embryo transfer emerges as the approach of choice for AHR to result in a full-term healthy newborn.

## Introduction

Since the first assisted reproductive technology (ART)-conceived infant was born in 1978, ART have developed and gained popularity worldwide. Thus, ART have increased notably the chances of pregnancy of subfertile couples [1]. At present, 2.6% of newborns in Europe [2], and more than 9% in Spain [3] are conceived by AHR.

In the early days of assisted reproductive technology, several fertilized eggs were transferred to increase the chances of pregnancy. Thus, the transfer of two, three or even four fertilized eggs was common practice [4]. This was routine practice in numerous countries in the 80s and 90s, as it was permitted by law. In Spain, Law 35/1988 on AHR did not establish a limit to the number of fertilized eggs to transfer, a decision that was left at physician’s discretion. This situation changed in 2003 with Law 45/2003, which only authorized the transfer of a maximum of three fertilized eggs per cycle. Since then, numerous studies have alerted on the dramatic increase in the rate of multiple pregnancies associated with ART, especially twin pregnancies, which accounted for 26% of multiple pregnancies [5]. Multiple pregnancies are the most frequent iatrogenic complications of ART and are a risk factor, as compared to singleton pregnancy. Thus, multiple pregnancy is associated with higher maternal mortality and morbidity rates and perinatal problems such as preterm birth, and low birthweight [6].

## Materials and methods

A literature search was performed on Entrez Pubmed (US National Library of Medicine, National Institute of Health; http://www.ncbi.nlm.nih.gov/pubmed/). The search terms used were infertility, *in vitro* fertilization (IVF), multiple embryo transfer, single-embryo transfer (SET), elective single-embryo transfer (eSET), multiple pregnancy, twin pregnancy, embryo cleavage blastocyst, vitrification, time-lapse technology, preimplantation genetic test and ART. After the primary search, other additional relevant articles were identified. A peer-review was performed of the 85 publications that met our inclusion criteria.

## European Union (EU) legal framework for embryo transfer

There is a common European legal framework for the use (procurement, storage, distribution and traceability) and testing of reproductive tissue and cells. Notably, a common legal framework has not been developed in the EU for the procedures performed in fertility centers [7], [8]. Therefore, each member state apply their own regulations which are regularly updated based on technical progress and the public or private coverage of treatment. In countries such as India, Japan or USA, ART are not regulated [9]. Nevertheless, most countries provide best practice guidelines designed with National or International Scientific Societies that complement national regulations.

With regard to embryo transfer, EU Members have established a limit to the number of embryos to transfer per attempt, although with a diversity of scenarios. In some countries, only one embryo can be transferred per cycle (Austria or Belgium in women younger than 36), whereas in other countries a maximum of three embryos can be transferred based on patient-physician preferences (Spain and Germany, although a maximum of two embryos is recommended in women younger than 37). In the majority of countries, although SET is recommended, there are age-dependent limits. In other countries such as France and Sweden, transferring more than two embryos is not permitted by law. In some countries such as Belgium, public coverage is contingent on embryo transfer policy [10]. In Bulgaria, the law establishes specific criteria including the age of the mother, number of failed attempts and embryo stage. At the far end is the Czech Republic, where a maximum number of embryos is not established by law, although most clinics recommend transferring one or two embryos.

## Scientific Society recommendations on the number of embryos to transfer

**Figure 1:**
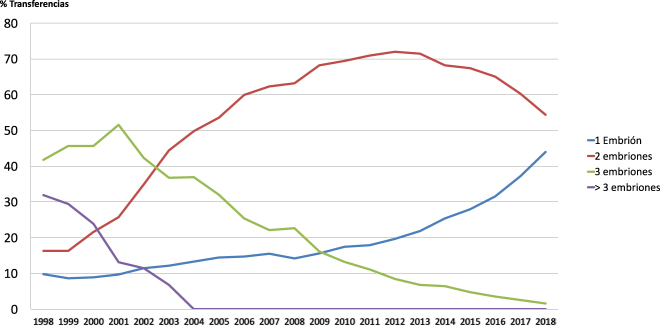
Evolution of fresh embryo transfer policies in Spain between 1998 and 2018, according to the 2018 National Registry of Activity—SEF Registry.

With the aim of reducing the rate of ART-related multiple pregnancies, the most relevant scientific societies have launched information campaigns to raise awareness on the effects of multiple embryo transfer. In 2002, the Spanish Society of Human Reproduction and Embryology (ESHRE) reviewed ART-associated risks and concluded that the purpose of *in vitro* fertilization (IVF) treatment is the birth of a single healthy infant; therefore, multiple pregnancy is but a complication of these treatments [11]. The Society for Assisted Reproductive Technology (SART) and the American Society for Reproductive Medicine (ASRM) started to raise awareness about the need to reduce the number of embryos to transfer. Indeed, SET accounted only for 1% of the total of embryo transfers performed in USA in 2002 [12], [13]. The same year, in Spain, fresh three-embryo transfer accounted for 42.3%, two-embryo represented 34.9%, and single-embryo transfer accounted for 11.4%, whereas these rates changed to 1.6, 54.4 and 44%, respectively in 2018 (Figure 1) [3], [5].

In relation to patients, the literature shows that twin pregnancy is widely accepted among patients, especially as infertility time and fertilization failures increase. Some studies, however, demonstrate that patient preference for multiple pregnancy decreases as their understanding of the associated risks increases, and acceptance of SET improves [14]. At present, awareness-raising campaigns and advances in laboratory technologies have resulted in eSET being offered as the best option in most fertility centers [15].

## Improvements and advances in eSET technologies

### Blastocyst culture

The best quality embryo is selected for eSET. Transfer can be performed at cell stage (2–3 days of development), morule stage (day 4), or blastocyst stage (days 5–6–7). However, laboratories increasingly opt for blastocyst transfer, as it guarantees the selection of the best embryo [16]. Blastocyst is the embryo structure with a highest cellularity and complexity obtained after laboratory culture fertilization and before it is transferred to the uterus. It is an advanced stage that has proven to be able to develop *in vitro* and differentiate into an *inner cell mass* that will give rise to the embryo and a *trophectoderm* that will develop into the placenta. This level of development, along with a qualitative evaluation of the embryo by morphology allow for the selection of the embryo with the best implantation potential [17]. Hence, culture of the embryo until the blastocyst phase, commonly known as long culture, is becoming more frequent. The implementation of this technique requires close control and monitoring of laboratory conditions and procedures. This is made possible by improvements in culture media and quality controls, and the development of time-lapse incubators [18].

The incubators traditionally used in the 80s regulated temperature and CO_2_, but they were only partially compartmentalized. This way, when incubators were opened, the conditions of all slices were altered. Temperature is set at 37 °C to simulate *in vivo* conditions, whereas CO_2_ control is essential to maintain an adequate pH in the culture medium (pH: 7.2–7.4) and ensure the correct development of the embryo [18], [19]. At present, the most widespread type of incubator is the so-called time-lapse incubators, where three gases are employed: CO_2_, O_2_ and N_2_. N_2_ reduces O_2_ concentrations to hypoxia levels (5%), as it occurs in the uterus, which has been proven to be essential for the development of the blastocyst [20]. In addition, in these incubators each slide is stored in a separate compartment, which minimizes that culture conditions are disturbed when the incubator is opened. These incubators, also known as benchtop incubators, provide optimal control and monitoring of gas concentrations and temperature.

### Embryo cryopreservation: vitrification

Vitrification/devitrification has also been a key to the spread of eSET programs. This procedure involves ultrarapid freezing/thawing by the exchange of water and cryoprotective substances, thereby preventing the formation of ice crystals, reaching survival rates of 78–100% [21]. This technique represents a turning point in ART, as slow freezing, which induces the formation of ice crystals inside cells, was the only technique available so far. This phenomenon causes embryo degeneration, with a survival rate of only 60%. On the other hand, the process is time-consuming and requires the use of more sophisticated equipment as compared to vitrification [22]. The high chances of live birth associated with vitrification facilitate eSET choice. Moreover, vitrification has allowed the spread of deferred frozen-instead of fresh-embryo transfer, which allows optimization of endometrial preparation and uterine environment, and increases the chances of success [23].

These technological advances help create optimal conditions for embryo development and cryopreservation *in vitro* and facilitate the selection of the embryo with the best implantation potential. This increases the chances of success of eSET, while multiple pregnancy rates are reduced. After the introduction of these changes in the laboratory, the criterion to calculate live birth rates is no longer based on the pregnancy rate per transfer but on “cumulative pregnancy rates”. The latter is based on all transfers of fresh or frozen embryos obtained from a single cycle of ovarian stimulation [23].

## Factors influencing eSET choice

### Medical contraindications to multiple embryo transfer

Although multiple pregnancy is also a risk factor for healthy women, the term mSET (*medical* SET) is reserved to women with total or relative contraindication to multifetal gestation for known medical conditions that may interfere with a good outcome, rather than for reasons associated with the fetus [6]. Elective SET is only *mandatory* in these circumstances. Women should be informed about these risks [6], [24]:

Absolute contraindications:

– Congenital uterine Müllerian anomaly, associated with a high risk for preterm birth.
– History of ruptured uterus.
– Cervical incompetence.
– Turner syndrome.
– Severe systemic disease.
– Severe psychiatric disease.
– Early multiple pregnancy loss.
– Insulin-dependent diabetes.

Relative contraindications:

– History of preterm labor in a singleton/twin pregnancy.
– Women without a couple.
– Couples formed by two women.
– Women of advanced age.
– Paraplegic women.
– Moderate/mild systemic disease.
– Moderate/mild psychiatric disease.
– Thromboembolic disease.

### Age of the patient

According to ASRM recommendations, eSET is the approach of choice in women with a good pregnancy prognosis. Thus, eSET is recommended for women younger than 35 on first or second cycle of ovarian stimulation, with a pregnancy in the previous cycle or egg receptors. Elective SET can also be considered in 35–40-year-old women with good quality blastocysts [25]. In women >40 years of age, the decision to transfer one or two blastocysts will depend on whether there are more than two transferrable/vitrificable blastocysts. Although the live birth rate doubles in elective two-blastocyst transfer, the rate of multiple birth increases from 0 to 22% [26]. In the 40–43 age range, age is not a predictive factor of the live birth rate, with a similar cumulative live birth rate in the two cases, but with a dramatic increase in the rate of multiple births (0 vs. 14.9%) [27]. However, previous recommendations about the number of embryos to transfer should also be adapted to the actual ovarian age, which may be enhanced or reduced [24].

In some countries, the number of embryos to transfer is regulated by national laws according to the age of the patient. Thus, we find legally enforced SET in Belgium, which regulates the number of embryos to transfer according to the age of the woman and the number of cycles completed. Multiple fresh embryo transfer is not permitted to women ≤36 years of age in the first or second cycle, except if low quality embryos are obtained in the second cycle. The government reimburses the costs of each cycle, but compliance is mandatory for all women undergoing a fertility treatment in Belgium even if they pay the treatment themselves [28].

### Embryo stage: Day 3 (D3) vs. Day 5 (D5)

The rationale for extending the time of culture and performing fresh blastocyst-stage transfer vs. D3 is that it improves uterine/embryo synchrony and allows for viable embryo self-selection, which results in better pregnancy and live birth rates [17, 29, 30]. However, there are no significant differences in multiple pregnancy rates, pregnancy loss rates, or cumulative pregnancy rates per cycle performed with blastocysts vs. D3 cleavage-stage embryos [17]. Additionally, high-quality cleavage-stage embryos (7–8 cells, minimal fragmentation, without multinucleation) show high implantation rates [31]. Thus, clinical pregnancy rates after the transfer of two good-quality cleavage-stage embryos are similar to that associated with elective single blastocyst transfer, but with the former being associated with higher twin pregnancy rates (43.26 vs. 0.6%) [32]. Therefore, the optimal day of transfer is contingent on the number and quality of embryos in D3.

Elective SET should also be considered in frozen cleavage-stage embryo transfer, especially after thawed blastocyst culture, as the rate of multiple pregnancy is significant, although lower than that of fresh embryo transfer.

In summary, eSET can be considered in the two embryo stages, taking into account couple sterility and embryo quality, and the performance of the cyropreservation program of the clinic in case of frozen embryo transfer [25]. Patients who desire a D5 transfer should be aware that they have more chances of no embryo being eventually available for transfer, and of a lower number of embryos being available for cryopreservation, since not all D3 embryos reach the blastocyst stage. Similarly, patients should be familiar with evidence of epigenetic alterations in long embryo culture in animal studies caused by the modulation of DNA methylation, which results in genetic imprinting defects [23].

### Number of good quality embryos

According to ASRM guidelines, eSET should be performed only in women with more than one high-quality embryo available and those with additional embryos for cryopreservation [25]. Double embryo transfer with one high-quality embryo plus one poor-quality embryo, at the blastocyst stage, does not increase the live birth rate but increases multiple births when compared with SET with a high-quality embryo during fresh or frozen embryo transfer treatment [33]. These results are consistent with previous studies and support the use of eSET when there is at least a high-quality embryo available [23], [34].

Controlled randomized studies demonstrate that fresh single top-quality blastocyst transfer is associated with a significantly higher ongoing pregnancy rate per cycle as compared to D3 embryo transfer [35], [36].

### Conventional morphologic embryo assessment vs. “Time-lapse”

The most widespread method for the selection of embryos is based on morphological evaluation and categorization according to factors such as cell number and symmetry, multinucleation, fragmentation and growth rate, to name a few. In Spain, the Association for the Study of Reproduction Biology (ASEBIR) has established a morphological classification system based on scientific evidence, expert opinion, external quality controls, and multicentric surveys and studies. This system associates a set of specific morphologic characteristics with an estimated likelihood of implantation [37]. On the other hand, assessment of embryo morphology could only be performed by direct observation under an inverted microscope. Although this method is simple, it only provides information of the embryo at the time of observation and is subject to interobserver variability, therefore, embryo evaluation is subjective [38]. The emergence of time-lapse ART technology represents a milestone in assisted reproduction. Time-lapse ART technology is based on a microscope equipped with a camera that allows continuous, noninvasive observation of embryos. This technology allows observation without having to take embryos out of the incubator, which disturbs culture conditions and may alter their development. Time-lapse incubators have made it possible to develop a more objective and reliable method for assessing embryo quality. Thus, the large amount of pictures taken provides thorough information about the morphological development of the embryo [39]. In addition, it offers the advantage that embryos can be selected on the basis of morphological, dynamic or morphokinetic criteria [19]. Therefore, time-lapse is especially useful for eSET, as it provides valuable data from continuous observation of embryonic development [40].

Numerous studies have demonstrated that embryo culture in time-lapse incubators does not affect their development as compared to conventional incubators [41]. In contrast, this new technology provides safe and stable culture conditions that favor embryonic development. A drawback to time-lapse incubator technology is the higher culture cost. However, the information obtained helps embryologist select the top-quality embryo with a higher probability of implantation by the use of predictive programs and algorithms based on more than 70 morphokinetic parameters. Only time-lapse technology enables observation of specific developmental events such as multinucleation, direct division (division from one to three cells) and cell fusion. Observing these events is crucial, as they are associated with a low implantation potential [42]. A plethora of studies have been performed to compare outcomes after embryo selection by morphokinetic criteria vs. specific morphologic observation. According to some authors, the morphokinetic approach improves implantation, pregnancy and live birth rate [43]. Other authors, however, have not found any significant relationship between improved outcomes and the use of time-lapse incubators [44]. There is not strong evidence that live birth rates are significantly higher in time-lapse culture and assessment vs. conventional methods. Therefore, the use of time-lapse technology to opt for eSET cannot be recommended [45].

### Preimplantation genetic testing for aneuploidy (PGT-A)

The rate of oocyte aneuploidy increases with maternal age and affects pregnancy and miscarriage rates [46]. PGT-A enables the selection of embryos with a correct set of chromosomes and exclude those with aneuploidy. This way, the rate of healthy live birth increases with the transfer of a single euploid embryo [47]. PGT-A requires the use of a safe technology that does not damage the embryo. The most extended technique today is blastocyst-stage trophectoderm biopsy. By this strategy, a higher number of cells are available for genetic testing, which improves the sensitivity of the technique. Thus, this technique significantly reduces the number of undiagnosed embryos (<5%) and does not require inner cell mass handling, which reduces risks. It also allows for the selection of euployd embryos with a better implantation potential even after the blastocyst is biopsied or vitrified one or two times [48]. Pregnancy rates do decrease with vitrification after a second embryo biopsy [48]. All this said, the guidelines where a specific number of embryos to be transferred are recommended are no longer based on maternal age, the number of embryos available or embryo stage, but also on the ploidy of the embryo [9], [46]. A variety of studies advocate for the use of PGT-A to increase the use of eSET in patients undergoing IVF [47], as the combination of the two approaches increases the live birth rate and reduces the multiple pregnancy rates [49]. There is evidence that PGT-A testing in women 35–40 years of age improves clinical and live birth rates, and mitigates the negative effects of maternal age on outcomes. However, it does not seem to improve cumulative live birth rates [50], [51]. Additionally, it has been observed that implantation, clinical pregnancy and live birth rates remain stable regardless of maternal age at eSET combined with PGT-A, and no significant differences have been observed with double embryo transfer [52]. Finally, it is important to consider that culture to blastocyst in time-lapse incubators combined with PGT-A-based embryo selection vs. conventional incubators significantly improves clinical pregnancy rates [53]. However, although PGT-A may benefit some groups of patients, there is no conclusive evidence available supporting its routine use as it increases treatment cost notably, especially in young women, and it is an invasive technique.

## SET live birth rates

### Comparative model: 2xeSET vs. 1xDET (*Double Embryo Transfer*)

Live birth rates associated with SET—whether it is elective or not-are usually lower (10–40%) than those expected after multiple-embryo transfer [54], [55]. In contrast, some authors report similar live birth rates for eSET to those obtained after nonelective multiple-embryo transfer [56]. There is also evidence that similar outcomes can be obtained in women of advanced age with a poorer prognosis [27]. Some authors, however, dispute the efficacy of SET in this group of patients [57]. On the other hand, there is enough evidence that the ongoing pregnancy and cumulative live birth rate are similar when a single cycle of double embryo transfer is compared with repeated SET [58]. Likewise, many studies show that SET helps reduce the high multiple pregnancy rates associated with multiple-embryo transfer (20–50%) to spontaneous multiple pregnancy rates (3%) [58], [59]. In conclusion, the efficacy of two SETs equals, or even improves with eSET, that of DET while avoiding he risks associated with multiple pregnancy.

## Cost-effectiveness of eSET

### Health costs of multiple pregnancy for the mother and her offspring

Multiple pregnancy is considered the main iatrogenic complication of ART, as it is associated with the occurrence of adverse events, which may affect both the mother and her offspring [13]. The main negative effects of multiple pregnancy are associated with preterm birth, fetal growth restriction, jaundice or respiratory complications. The probability of preterm labor is 5–9 times higher in multiple pregnancy as compared to singleton pregnancy [60]. An increase has been described in the incidence of maternal complications, including pre-eclampsia, gestational diabetes, placenta previa, placental detachment, premature rupture of membranes and caesarean section [60], [61], [62]. These complications may have more severe effects in women of advanced age [63]. Thus, multiple pregnancy is a known risk factor of maternal and fetal morbidity and mortality.

### Economic, social and health costs of multiple pregnancy

According to the literature, SET requires a higher number of embryo transfers to achieve pregnancy, as compared to multiple embryo transfer [58]. This involves a higher cost of ART: more hormone stimulation, embryo transfers and vitrification/thawing cycles. In addition, the costs of multiple pregnancy and delivery are 2–7 higher than those of singleton ones [64]. If pediatric care during the first year of life is included, the economic cost of multiple delivery is 20 times higher than singleton delivery [65].

ART costs are borne either by the national health system or by the patients themselves, which influences the choice for an embryo transfer strategy. Multiple pregnancies also involve additional costs in the long-term including clothing, car seats, food, and schooling, to name but a few [55]. After an analysis of costs and outcomes of DET vs. eSET, Fiddelers et al. [66] concluded that DET involves a higher cost from the economic point of view. Therefore, eSET emerges as the most cost-effective strategy, when more than one embryo transfer cycle is considered [66].However, it is worth noting that multiple pregnancy rate after multiple-embryo transfer decreases as the age of the mother increases, so this approach can be considered cost-effective in women of advanced age [67].

In conclusion, multiple pregnancies have a high social and health cost, which can be reduced with repeated SET without it affecting ART effectiveness, if we consider cumulative live birth rates [68].

### Psychosocial costs of multiple pregnancy for patients

There are some psycho-social factors associated with pregnancy and delivery that should be considered. According to some population-based studies, women are more likely to have depression and episodes of postpartum stress after multiple delivery, as compared to singleton delivery. Nevertheless, no significant differences were observed in the probability to experience these symptoms following assisted and natural conception [69].

Likewise, some authors associate multiple birth with decreased marital satisfaction, loss of productivity, and a lower probability of the mother to have a paid job [55], [64]. These data support the need to reinforce psychological support to parents of multiples.

## Elective SET implementation

### Education and counseling

Despite the risks associated with multiple pregnancy and delivery, up to 80% of patients’ desire a multiple-embryo transfer as the first option [55]. The main reasons include unawareness of the associated risks, desire to maximize the chances of success, or reduce the cost of ART, or the idea to achieve a multiple birth in a single attempt. Informing patients on cumulative live birth rate, health risks, and economic and emotional implications of multiple births has been proven to be effective in increasing the choice of SET [14]. The provision of information from personalized prediction tools that estimate the chances of IVF success has also demonstrated to be useful in helping patients make decisions about IVF, as they are perceived as an objective source of information [55].

### Economic and social incentive policies

Some countries such as Turkey, Sweden, Denmark, Belgium, New Zealand or Canada have established or promoted a national SET policy. The purpose of these policies is twofold: to avoid health risks for the mother and the newborn, and reduce the high health costs associated with multiple births. Measures range from restrictions to multiple-embryo transfers for most patients to limitations to the number of embryos transferred for patients to the ART process to be partially or totally borne by the national health system. These countries have achieved to increase SET rates and reduce multiple births to 80 and 5%, respectively, in the case of Australia, whereas cumulative clinical pregnancy and live birth rates have remained stable [55].

This evidence demonstrates that educational interventions along with SET incentive policies in women with a good prognosis and public funding are effective in promoting the choice for SET in countries as culturally distant as USA, Japan and New Zealand [14]. Economic stimuli include public funding, total/partial coverage of ART procedures by insurance providers, or promotional campaigns of AHR centers, which may include cryopreservation and/or frozen embryo transfer at free cost. However, it is worth noting that SET incentive measures do not seem to be as effective in countries where public funding is available for ART treatments, as it is the case of Denmark and Sweden [14].

## Conclusions

More than seven million children have been born in the world as a result of assisted conception. Since the early days of ART, high multiple pregnancy rates have posed a difficult dilemma due to the higher risk for preterm labor and its higher associated maternal and fetal morbidity and mortality rates.

In the recent years, significant advances have been made in the field of reproductive medicine, which implementation has resulted in a significant increase in live birth rates. Special attention needs to be paid on AHR laboratory innovations such as culture to blastocyst stage, cryopreservation by vitrification, time-lapse technology or PGT-A. These techniques favor the SET application, even in patients with a poor prognosis, without it affecting live birth rates.

Despite continuous progress in the field of ART, potentially significant differences will be observed between SET and multiple-embryo transfer in pregnancy and live birth rates as long as outcomes are expressed as rates per embryo transfer. Cumulative rates are more precise, as they include fresh and frozen embryo transfers of embryos obtained from the same cycle.

This review is aimed at raising awareness among ART professionals about the impact that AHR-related multiple pregnancy has on public health and promoting SET as the strategy of choice for having a healthy full-term newborn at home.
